# Regulation of ASPP2 Interaction with p53 Core Domain by an Intramolecular Autoinhibitory Mechanism

**DOI:** 10.1371/journal.pone.0058470

**Published:** 2013-03-05

**Authors:** Shahar Rotem-Bamberger, Chen Katz, Assaf Friedler

**Affiliations:** Institute of Chemistry, The Hebrew University of Jerusalem, Givat Ram, Jerusalem, Israel; University of South Florida College of Medicine, United States of America

## Abstract

ASPP2 is a key protein in regulating apoptosis both in p53-dependent and-independent pathways. The C-terminal part of ASPP2 contains four ankyrin repeats and an SH3 domain (Ank-SH3) that mediate the interactions of ASPP2 with apoptosis related proteins such as p53, Bcl-2 and the p65 subunit of NFκB. p53 core domain (p53CD) binds the n-src loop and the RT loop of ASPP2 SH3. ASPP2 contains a disordered proline rich domain (ASPP2 Pro) that forms an intramolecular autoinhibitory interaction with the Ank-SH3 domains. Here we show how this intramolecular interaction affects the intermolecular interactions of ASPP2 with p53, Bcl-2 and NFkB. We used biophysical methods to obtain better understanding of the relationship between ASPP2 and its partners for getting a comprehensive view on ASPP2 pathways. Fluorescence anisotropy competition experiments revealed that both ASPP2 Pro and p53CD competed for binding the n-src loop of the ASPP2 SH3, indicating regulation of p53CD binding to this loop by ASPP2 Pro. Peptides derived from the ASPP2-binding interface of Bcl-2 did not compete with p53CD or NFkB peptides for binding the ASPP2 n-src loop. However, p53CD displaced the NFκB peptide (residues 303–332) from its complex with ASPP2 Ank-SH3, indicating that NFκB 303–332 and p53CD bind a partly overlapping site in ASPP2 SH3, mostly in the RT loop. These results are in agreement with previous docking studies, which showed that ASPP2 Ank-SH3 binds Bcl-2 and NFκB mostly via distinct sites from p53. However they show some overlap between the binding sites of p53CD and NFkB in ASPP2 Ank-SH3. Our results provide experimental evidence that the intramolecular interaction in ASPP2 regulates its binding to p53CD and that ASPP2 Ank-SH3 binds Bcl-2 and NFκB via distinct sites.

## Introduction

p53 is one of the major tumor suppressor proteins in the cell. It is a transcription factor that induces growth arrest or apoptosis in response to cellular stress [1], [2]. The p53 response depends on the amount and type of cellular stress: In cells under low stress, p53 functions as a protector and its activation leads to cell cycle arrest and DNA repair. When the stress is high, p53 induces senescence or apoptosis that kills the cell in order to save the organism [2]. Following its induction, p53 binds specific promoters in the DNA and activates the transcription of a wide array of target genes, aimed at eliminating the danger of potential cancer [3]–[5]. p53 contains several structural and functional domains: an N-terminal transactivation domain followed by a proline rich domain, a DNA-binding/core domain and an oligomerization domain in its C-terminus [6].

**Figure 1:**
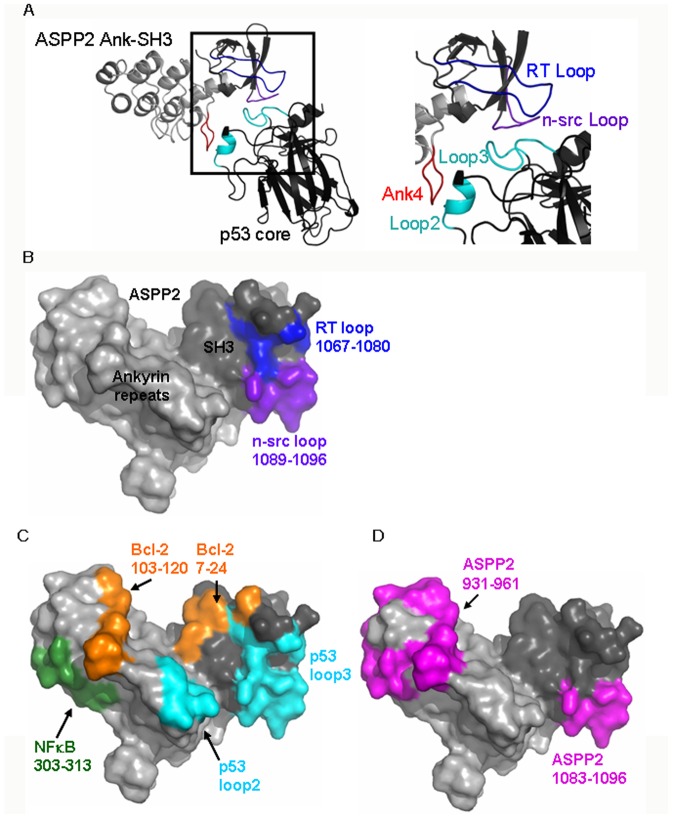
The ASPP2 Ank-SH3 – p53CD complex PDB ID: 1YCS [14]. (A) Crystal structure of the complex between p53CD (black) and ASPP2 927–1119 (gray). The RT loop (residues 1067–1080, blue) and the n-Src loop (residues 1089–1096, purple) in the ASPP2 SH3 domain bind the L3 loop of p53 (residues 241–249, cyan), while the fourth ankyrin repeat (residues 1020–1027, red) binds the L2 loop (residues 178–183, cyan) of p53CD [14]; (B) The structure of the four ankyrin repeats (light gray) and the SH3 domain (dark gray) of ASPP2. The p53CD binding sites in the ASPP2 SH3 domain of ASPP2 are shown with the RT loop in blue and the n-src loop in purple. (C) The binding sites of ASPP2 Ank-SH3 to p53CD (cyan), Bcl2 (orange) and NFκB (green) are all on the same face of the molecule but with minimal overlap between the binding sites [21]; (D) The ASPP2 Pro (residues 693–918) binding sites in ASPP2 Ank-SH3 (magenta) [13].

ASPP2 is one of the three members of the ASPP (Apoptosis Stimulating Proteins of p53) family, which also includes ASPP1 and iASPP. ASPP1 and ASPP2 activate the apoptotic p53 response, but not the cell-cycle arrest response, while iASPP inhibits p53-mediated apoptosis [7], [8]. ASPP2 gene methylation causes low levels of ASPP2 expression in human cancers, which is correlated with poor clinical outcome [7], [9], [10]. Bbp (bcl-2 binding protein) is an ASPP2 variant lacking the 123 N-terminal residues. Both ASPP2 and Bbp are encoded by the TP53BP2 gene [8], [11]. ASPP2 contains several structural and functional domains: Its N-terminus (residues 1–83) has the structure of a beta-Grasp ubiquitin-like fold [12]. It is followed by a predicted alpha-helical domain located between residues 123–323 [11], and an intrinsically disordered proline-rich domain (ASPP2 Pro) between residues 674–902 [13]. The C-terminal part of ASPP2 contains four ankyrin repeats and an SH3 domain (ASPP2 Ank-SH3), as observed in its crystal structure in complex with p53CD (Figure 1A) [8], [11], [14]. p53CD loop2 (residues 164–194) and loop3 (residues 237–250) bind ASPP2 in the loop of the fourth ankyrin repeat (residues 1020–1026), the n-src (residues 1089–1096) loop and the RT loop (residues 1067–1080) in the SH3 domain (Figure 1A and B and C) [14], [15]. Besides p53, the Ank-SH3 domains of ASPP2 mediate its interactions with numerous partner proteins such as Bcl-2 and the p65 subunit of NFκB, most of which are also involved in apoptosis or its regulation [11], [16], [17]. NFκB is a transcription factor that is activated following a wide array of signals and induces genes that can protect the cell or contribute to apoptosis [18], [19]. The antiapoptotic Bcl-2 protein belongs to the Bcl-2 family that contains proapoptotic and antiapoptotic members, which form homo-/heterodimers that maintain the balance between apoptosis and cell survival [20]. Structural models for the interactions of ASPP2 Ank-SH3 with Bcl-2 and NFκB suggest that Bcl-2 103–120 and NFκB 303–313 bind two different non-overlapping sites in the first ankyrin repeat of ASPP2 between residues 931–961. Bcl-2 7–24 also binds next to the RT loop of the SH3 domain (Figure 1C) [21], [22]. We have previously demonstrated an intramolecular interaction between ASPP2 Pro and ASPP2 Ank-SH3: ASPP2 Pro (residues 693–918) binds the first ankyrin repeat (residues 931–961) and the n-src loop of the SH3 domain (residues 1083–1096) (Figure 1D) [13]. This intramolecular interaction regulates the intermolecular protein-protein interactions of ASPP2 by an autoinhibitory mechanism, as shown for a peptide derived from NFκB *in vitro* and for the protein Yap *in vivo* [13], [23]. The sites in ASPP2 that bind Bcl-2 and NFκB were also identified by us [13], [21], [22], indicating that Bcl-2, NFκB and p53 all bind the same interface of ASPP2 Ank-SH3 but their binding sites do not overlap.

The *TP53* gene encoding p53 is frequently mutated in human cancers and most of these reported mutations are in the DNA-binding core domain of the protein (p53CD) [24], [25]. Some of these mutations lower the thermodynamic stability of p53, resulting in its unfolding and inactivation [26]. A nine-residue peptide derived from ASPP2 n-src loop (residues 1089–1097), also termed CDB3, restored the specific DNA binding activity of the highly destabilized p53CD mutant I195T to the levels similar to the wild-type level [27]. This peptide slowed down the unfolding of p53CD at 37°C in a concentration-dependent manner by maintaining the mutant protein in a folded conformation and preventing its aggregation, therefore allowing it enough time to reach the nucleus and bind its sequence-specific target DNA or the p53 binding proteins that stabilize it [27]. This ASPP2 n-src loop peptide rescued the structural effects of mutation in p53 R249S mutant back towards the wild-type-like structure [28] and activated other mutants p53 in cells [29].

Here we show, using fluorescence anisotropy competition experiments, that the intramolecular interaction in ASPP2 regulates the binding of ASPP2 Ank-SH3 to p53CD. The ASPP2 SH3 domain bound ASPP2 Pro and p53CD via the same site. p53CD binding to the ASPP2 SH3 n-src peptide (ASPP2 1089–1097) was inhibited by the ASPP2 Pro peptide (residues 722–737). p53CD displaced the ASPP2 Pro peptide from its complex with ASPP2 Ank-SH3. ASPP2-binding peptides derived from Bcl-2 and NFκB did not compete with each other or with p53CD for its binding to the ASPP2 n-src peptide. However, p53CD displaced the NFκB peptide from its complex with ASPP2 Ank-SH3. These results strongly support our previous docking studies [21], [22]. They provide experimental evidence that the intramolecular domain-domain interaction in ASPP2 regulates its binding to p53CD and that ASPP2 Ank-SH3 binds Bcl-2 and NFκB via distinct sites.

## Materials and Methods

### Expression and purification of p53CD

The pRSET A (Invitrogen) vector containing p53 94–312 (kind gift from Prof. A. R. Fersht, MRC-LMB Cambridge), was transformed into *E. coli* strain C41 (DE3) (Lucigen). Cells were grown at 37°C in 2×YT medium to an optical density (600 nm) of ∼0.6 and induced with 1 mM isopropyl-β-D-1-thiogalactoside (IPTG) and 100 µM ZnSO_4_ were added upon induction. Cells were harvested after 20 h of incubation at 20°C and were lysed by Microfluidizer (Microfluidics) in 20 mM phosphate buffer pH 7.2, 25 mM NaCl, 10 mM β mercaptoethanol (βME), 0.2 mM PMSF and 1∶200 protease inhibitor P-8849 (Sigma). Soluble lysate was loaded onto a HiTrap SP 5 ml cation exchange column (Pharmacia) and eluted with NaCl gradient (0–1 M) using an FPLC system (ÄKTA explorer, Amersham Biosciences). The eluted protein was diluted to a final salt concentration <50 mM and purified on a HiTrap heparin 5 ml column (Pharmacia) in 20 µM phosphate buffer pH 7.2, 25 mM NaCl, 10 µM βME with NaCl gradient (0–1 M) for elution. The eluted protein was further purified by gel filtration on a Superdex 75 column (100×1.6 cm; Amersham Biosciences). The protein was stored in 20 mM phosphate buffer pH 7.2, 300 mM NaCl, 10 mM βME.

### Expression and Purification of ASPP2 Ank-SH3

pRSET HLT ASPP2 893–1128 vector (a kind gift from Prof. A. R. Fersht, MRC-LMB Cambridge) was expressed, and the protein was purified as described [13].

### Peptide Synthesis and Purification

Peptides were synthesized on a Liberty Microwave-Assisted Peptide Synthesizer (CEM), using standard Fmoc chemistry as described [30]. Peptides were fluorescein-labeled at the N terminus as described [30]. For peptides sequences see Table 1.

**Table 1:** The ASPP2 Ank-SH3 binding peptides used in this study.

Peptide	Sequence
ASPP2 722–737	*WRKKLSNAPRPLKKRSS
ASPP2 1089–1097	REDEDEIEW
NFκB 303–332	KRTYETFKSIMKKSPFSGPTDPRPPPRRIA
Bcl-2 7–24	YDNREIVMKYIHYKLSQR
Bcl-2 103–120	RRYRRDFAEMSSQLHLTP

* Trp was added at the N terminus of the peptide for UV spectroscopy.

### Fluorescence Anisotropy

Measurements were performed at 10°C by using a PerkinElmer Life Sciences LS-50b spectrofluorometer equipped with a Hamilton microlab M dispenser. Titration of ASPP2 Ank-SH3 into the fluorescein-labeled (Fl) peptides was performed in 20 mM Hepes at pH 7.0 at ionic strength (IS) of 50 mM, 100 mM or 150 mM NaCl and 5 mM βME. Titration of p53CD, ASPP2 722–737 into the Fl-ASPP2 1089–1097 were performed in 50 mM Hepes at pH 7.0 10 mM NaCl and 10 mM βME as described [27] or in 20 mM Hepes at pH 7.0 at IS of 50 mM by NaCl, and 5 mM βME for NFκB 303–332. Fluorescence was measured with excitation at 480 nm and emission at 530 nm. The labeled peptide was placed in the cuvette in a volume of 1 ml, at a concentration of 100 nM, and 100–200 µl of titrant were placed in the dispenser. Additions of 5–10 µl of protein or unlabeled peptide were titrated at 60–90 sec intervals, the solution was stirred for 10 seconds, and the fluorescence and anisotropy were measured. Dissociation constants (*K_d_*) were calculated as described in ref. [13] by fitting the anisotropy titration curves (corrected for the dilution effect) using Kaleidagraph (Synergy Software, Reading, PA) to the following equation describing a 1∶1 binding model.
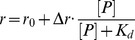
Where *r* is the measured fluorescence anisotropy value, *Δr* is the amplitude of the fluorescence anisotropy change from the initial value (peptide only) to the final value (peptide in complex), *r*_0_ is the starting anisotropy value corresponding to the free peptide, [*P*] is the protein concentration, *K*_d_ is the dissociation constant.

In the competition experiments, the conditions were as follows: 100–200 µl of competitor peptide or protein were added to a 1200 µl mixture of 83 nM Fl-ASPP2 722–737 and 8.3 µM ASPP2 Ank-SH3 at 20 mM Hepes pH 7.0, IS = 50 mM, 5 mM βME; 200 µl of 130 µM of competitor peptide were added to a 1150-µl mixture of 87 nM Fl peptide ASPP2 1089–1097 and 6.5 µM p53CD at 50 mM Hepes pH 7.0, IS = 20 mM, 10 mM βME; 200 µl of 130 µM of Bcl-2 103–120 or Bcl-2 7–24 were added to a 1100 µl mixture of 87 nM Fl-NFκB 303–332 and 2.25 µM ASPP2 Ank-SH3 or to 1150 µl mixture of 87 nM Fl-ASPP2 722–737 and 4.5 µM ASPP2 Ank-SH3 at 20 mM Hepes pH 7.0, IS = 50 mM, 5 mM βME. The *K*_d_ of the competitor was calculated as described [27].

## Results

### p53CD and ASPP2 Pro share the same binding site in ASPP2 SH3

**Figure 2:**
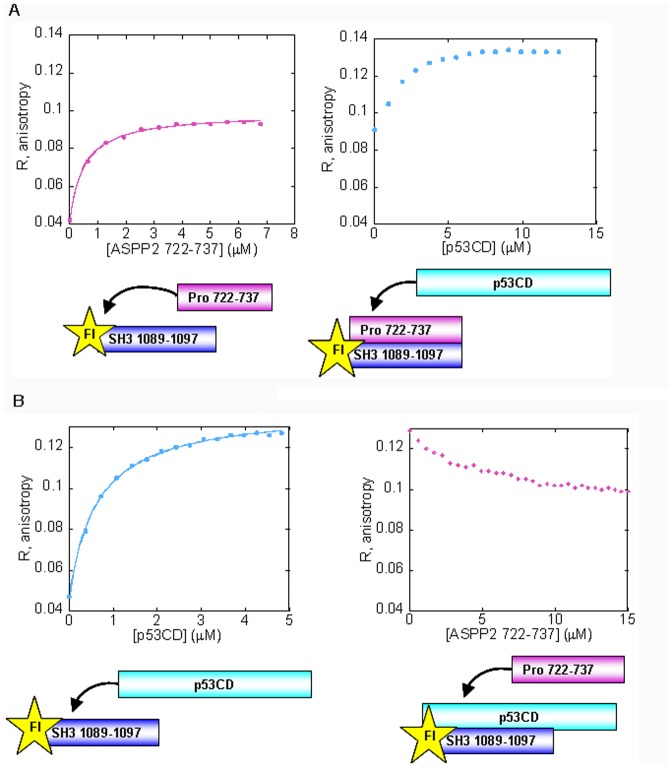
p53CD and ASPP2 Pro 723–737 compete for binding the Fl-ASPP2 n-src loop peptide residues 1089–1097: fluorescence anisotropy experiments. (A) A peptide derived from the proline rich domain of ASPP2 residues 722–737 binds the ASPP2 n-src peptide with *K*_d_ = 0.50±0.03 µM (magenta); Following titration of p53CD (cyan) into to the fully formed complex between Fl-ASPP2 1089–1097 and ASPP2 722–737, the anisotropy increased (B) p53CD residues 94–312 bind the ASPP2 n-src peptide with *K*_d_ = 0.63±0.02 µM (cyan). Unlabeled ASPP2 722–737 (magenta) displaced p53CD form its fully formed complex with ASPP2 1089–1097.

A major p53-interacting site in ASPP2 SH3 is situated in the n-src loop between residues 1089–1097 [14], [27] (Figure 1A, B). We have previously shown by peptide array screening that the n-src peptide also binds ASPP2 Pro [13]. Following this observation we tested whether the ASPP2 intramolecular interaction regulates the binding of ASPP2 Ank-SH3 to p53CD by performing a series of fluorescence anisotropy competition experiments. The ASPP2 Pro peptide (residues 722–737) was titrated into the fluorescein-labeled (Fl) ASPP2 n-src peptide (residues 1089–1097). The peptides bound each other with *K*_d_ = 0.50±0.03 µM (Figure 2A). p53CD bound Fl-ASPP2 n-src peptide with *K*_d_ = 0.63±0.02 µM (Figure 2B) in agreement with previously reported studies [27], [29]. In the competition assay, non-labeled ASPP2 Pro peptide (722–737) was added to the fully formed complex between p53CD and the Fl-n-src peptide. The non-labeled ASPP2 Pro peptide (722–737) displaced p53CD from its complex with Fl-n-src peptide (Figure 2B), indicating an overlapping binding site. The anisotropy decreased only mildly because the ASPP2 pro peptide bound the Fl-n-src peptide instead of p53CD. In the opposite experiment p53CD was added to a pre-formed complex between the ASPP2 Pro peptide and the Fl-ASPP2 n-src peptide (Figure 2A). In this case the final anisotropy level increased, because the heavier p53CD molecule (MW∼24 kDa) bound the Fl-n-src peptide instead of the lighter ASPP2 Pro peptide (1.7 kDa). In this competition assay p53CD and the Pro peptide did not bind each other but rather both bound the fluorescein labeled n-src peptide and displaced each other from the complex.

**Figure 3:**
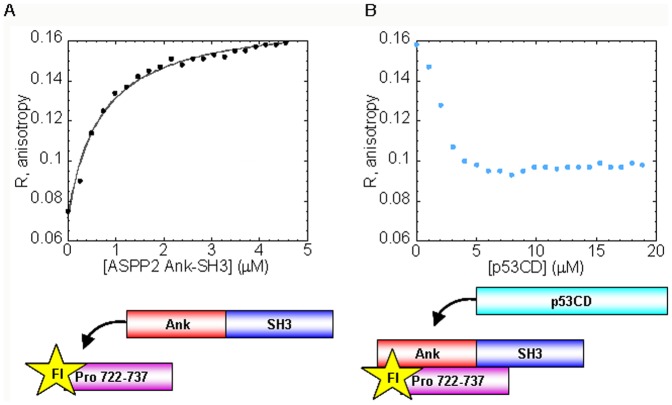
p53CD and the Fl-ASPP2 Pro 722–737 peptide compete for binding ASPp2 Ank-SH3. (A) ASPP2 Ank-SH3 binds ASPP2 Pro 722–737 with *K*_d_ = 0.68±0.07 µM. (B) p53CD was titrated into the fully formed complex between ASPP2 Ank-SH3 and Fl-ASPP2 722–737 and displaced the Fl-ASPP2 722–737 peptide.

To test whether p53CD binding interferes with the domain-domain interaction in ASPP2, ASPP2 Ank-SH3 was titrated into Fl-ASPP2 Pro peptide (Figure 3A) and ability of p53CD to displace it from the complex was tested (Figure 3B). p53CD displaced ASPP2 Pro peptide from its complex with ASPP2 Ank-SH3, in agreement with our results above. In this competition assay p53CD bound ASPP2 Ank-SH3 and displaced the fluorescein labeled Pro peptide, resulting in *K*_d_ = 0.47 µM for the p53CD – ASPP2 Ank-SH3 at IS = 50 mM.

### Bcl2 and NFκB derived peptides did not compete with p53CD for binding the ASPP2 SH3 n-src loop

**Figure 4:**
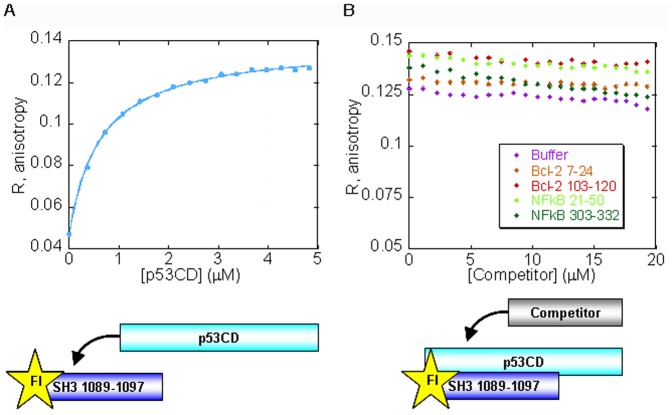
Bcl-2 and NFκB peptides bind the ASPP2 n-src loop 1089–1097 peptide in a different site than p53CD. (A) p53CD binds Fl-ASPP2 1089–1097 ASPP2 SH3 derived peptide In fluorescence anisotropy experiments with *K*_d_ = 0.63±0.02 µM (B) Non-labeled Bcl-2 or NFκB peptides were titrated into the fully formed complex between Fl-ASPP2 1089–1097 and p53CD. None of the peptides displaced p53CD from the complex.

It is known from our previous theoretical studies that ASPP2 Ank-SH3 binds p53CD, Bcl2 and NFκB 1–313 via the same face of the molecule but with minimal overlap between the binding sites (Figure 1C) [31]. To test this experimentally we used fluorescence anisotropy competition experiments. p53CD was titrated into Fl-ASPP2 n-Src loop (residues 1089–1097) (Figure 4A) and unlabeled peptides, derived from the ASPP2-binding sites in Bcl-2 or NFκB, were added to the fully formed complex (Figure 4B). The peptides Bcl-2 7–24, Bcl-2 103–120 [22], NFκB 21–50 and NFκB 303–332 [13], [21] (Table 1) did not compete with p53CD on binding the ASPP2 peptide, indicating that the binding sites are indeed different and providing an experimental evidence for our prediction.

**Figure 5:**
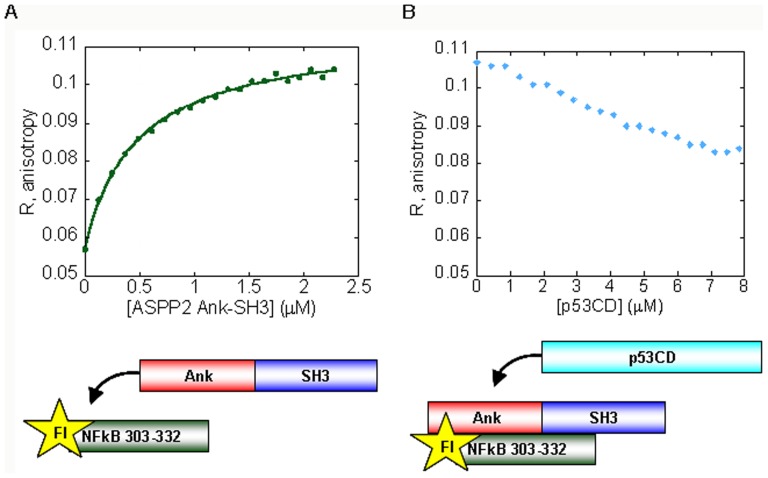
p53CD displaces NFκB 303–332 from its complex ASPP2 Ank-SH3. (A) Binding of ASPP2 Ank-SH3 to Fl-NFκB 303–332 resulting in K*_d_* = 0.47±0.02 µM; (B) p53CD displaced ASPP2 Ank-SH3 from its fully formed complex with Fl-NFκB 303–332.

Fl-NFκB 303–332 was previously shown to bind ASPP2 Ank-SH3 and compete with the Pro peptide ASPP2 722–737 [13]. We thus assumed that NFκB 303–332 also binds the SH3 domain of ASPP2, similar to p53CD and the ASPP2 Pro peptide. To test this we titrated p53CD into the fully formed complex between ASPP2 Ank-SH3 and Fl- NFκB 303–332 (Figure 5A). p53CD displaced the NFκB peptide from the complex (Figure 5B), indicating that p53CD and NFκB 303–332 share a partly overlapping binding site in the ASPP2 SH3 domain. *K*_d_ for the p53CD – ASPP2 Ank-SH3 interaction in this experiment (IS = 150 mM) was found to be 2.45 µM, in agreement with the previously reported ITC results performed at similar condition [32].

### Bcl2 derived peptides and the ASPP2 Pro peptide do not share the same binding site in ASPP2 Ank-SH3

**Figure 6:**
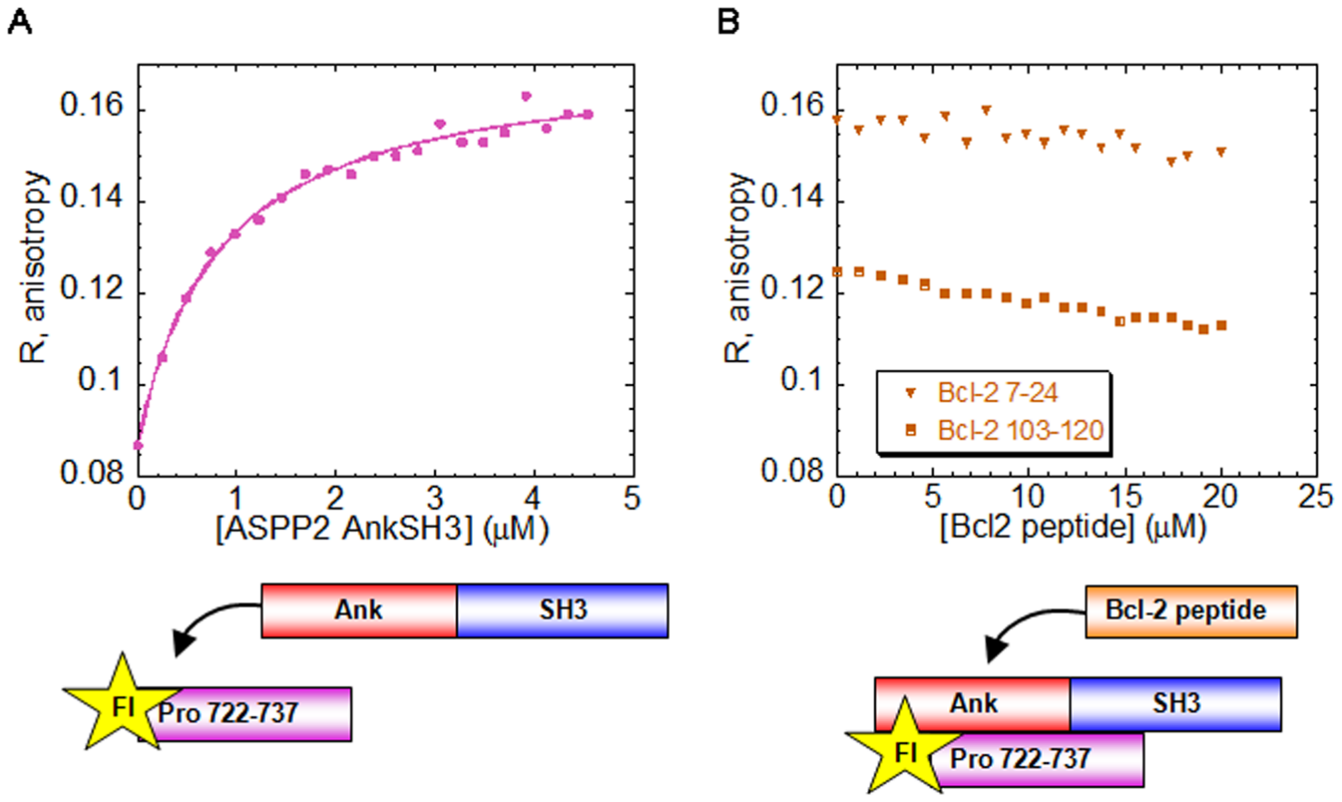
ASPP2 Ank-SH3 binds ASPP2 722–737 and is not displaced by Bcl-2 peptides. (A) ASPP2 Ank-SH3 binds Fl-ASPP2 722–737 with K*_d_* = 0.82±0.08 µM (magenta); (B) Non-labeled Bcl-2 103–120 (orange square) or Bcl-2 7–24 (orange triangle) peptides were titrated into the fully formed complex between Fl- ASPP2 722–737 and ASPP2 Ank-SH3 but did not compete with ASPP2 Ank-SH3 on peptide binding.

We have previously shown that p53CD, the NFκB peptide 303–332 and ASPP2 Pro 722–737 [13] bind the same site in ASPP2 Ank-SH3 domains. To test if Bcl-2 7–24 and Bcl-2 103–120 also share the same binding site we titrated Bcl-2 peptides into the complex between ASPP2 Ank-SH3 and Fl-ASPP2 722–737 (Figure 6A). Both Bcl-2 peptides did not displace Fl-ASPP2 722–737 (Figure 6B) from the complex, in agreement with our previous results showing that the Bcl-2 binding sites are not in the n-src loop of ASPP2 (Figure 1B and C).

### Bcl-2 and NFκB peptides do not share the same binding site in ASPP2

**Figure 7:**
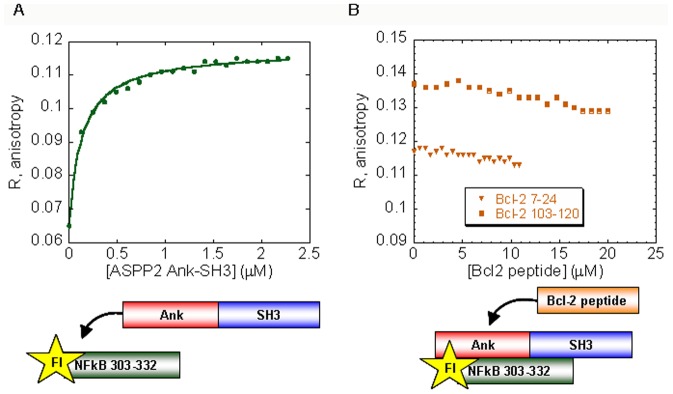
ASPP2 Ank-SH3 binds NFκB 303–332 and is not displaced by Bcl-2 peptides. (A) ASPP2 Ank-SH3 binds Fl-NFκB 303–332 with K*_d_* = 0.13±0.01 µM; (B) Non-labeled Bcl-2 103–120 (orange square) or Bcl-2 7–24 (orange triangle) peptides were titrated into the fully formed complex between Fl-NFκB 303–332 and ASPP2 Ank-SH3 but did not compete with ASPP2 Ank-SH3 on peptide binding.

According to our structural models, Bcl-2 103–120 and NFκB 303–313 bind two different non-overlapping sites in the first ankyrin repeat of ASPP2 between residues 931–961 (Figure 1C) [21], [22], [31]. Here we showed that NFκB 303–332 and p53CD share a partly overlapping binding site in the ASPP2 SH3 domain, which is not predicted to overlap the Bcl-2 7–24 binding sites in the SH3 domain (Figure 1C) [22]. To test this experimentally, we performed fluorescence anisotropy competition experiments between the ASPP2 Ank-SH3-binding peptides Bcl-2 7–24, Bcl-2 103–120 and NFκB 303–332. ASPP2 Ank-SH3 was titrated into Fl-NFκB 303–332 (Figure 7A), and the ability of the non-labeled peptides Bcl-2 103–120 and Bcl-2 7–24 to displace it was tested. The Bcl-2 peptides did not displace NFκB 303–332 from the complex with ASPP2 Ank-SH3 (Figure 7B). These results indicate that the Bcl-2 and NFκB peptides bind ASPP2 Ank-SH3 at different sites and validate the structural models [21], [22], [31].

## Discussion

Here we used fluorescence anisotropy competition experiments to reveal the effect of the intramolecular interaction in ASPP2 on its binding to p53CD. The change in anisotropy in the competition experiments can result from two reasons: (i) interaction of the unlabelled competitor with the labeled peptide, releasing the partner protein. In such a case the anisotropy decreases because the new complex formed between two peptides is smaller than the peptide-protein complex. This was the case when p53CD was titrated into the complex between the Fl-n-src peptide and the ASPP2 Pro peptide; (ii) interaction of the unlabelled competitor with the protein, releasing the labeled peptide. Here the anisotropy decreases because the labeled peptide is released from its complex with the partner protein. This was the case when p53CD was titrated to the complex between Fl-ASPP2 Pro peptide and ASPP2 Ank-SH3.

### p53CD and a peptide derived from ASPP2 Pro bind ASPP2 SH3 at the same site: Biological consequences

In this study we found that p53CD and the ASPP2 Pro 722–737 peptide compete for this same binding site in the ASPP2 SH3 domain. This suggests that the intramolecular interaction in ASPP2, between the Ank-SH3 and Pro domains, has a role in regulating the interactions of ASPP2 with p53. The ASPP2 n-src peptide (ASPP2 residues 1089–1097) was shown to sensitize colorectal carcinoma HCT116 cells, but not HCT116 p53−/− cells, to apoptosis induced by γ-irradiation [29]. It is possible that the peptide inhibits the intramolecular interaction in ASPP2 and thus activates the p53-ASPP2 interaction that induces apoptosis. The n-src peptide also restored the wild-type conformation to R175H and R273H mutant p53 in cells [29]. This effect may be due to the induction of the p53 interaction with ASPP2.

### Bcl-2 peptides do not displace NFκB peptide and Pro peptide from ASPP2 Ank-SH3

We previously suggested, based on computational models, that ASPP2 Ank-SH3 binds NFκB and Bcl-2 in different non-overlapping sites (Figure 1C) [31]. In the computational model the Bcl2 BH4 derived peptide Bcl-2 7–24 binds next to the SH3 RT loop of ASPP2 while Bcl-2 103–120 binds the first ankyrin repeat of ASPP2 [22]. The NFκB 303–332 peptide is also predicted to bind ASPP2 Ank-SH3 via the same sites [21]. Here we showed by fluorescence anisotropy competition experiments that indeed NFκB 303–332 does not compete with Bcl-2 derived peptides for binding ASPP2 Ank-SH3. The results provide experimental evidence to the models [21], [22] according to which NFκB and Bcl-2 both bind the first ankyrin repeats of ASPP2 but binding is mediated via different residues. The binding sites may have a little overlap but the affinity of the NFκB 303–332 peptide to the ASPP2 Ank-SH3 domain may be high enough (Figure 6A) so that the low affinity Bcl-2 peptides cannot displace it. Indeed, ASPP2 Ank-SH3 binds Fl-Bcl-2 7–24 with *K*_d_ = 4.7±0.2 µM [22] but its affinity to Fl-NFκB 303–332 is 0.13±0.01 µM under the same conditions.

The Bcl-2 peptides did not compete with the ASPP2 Pro peptide for binding ASPP2 Ank-SH3. According to our previously published computational model the, the n-src loop of the ASPP2 SH3 domain is not involved in Bcl-2 binding (Figure 1B, C).

Here we identified the specific binding site of the Pro peptide to the n-src loop (Figure 2A). The full length ASPP2 Pro domain, residues 693–918, also binds the first ankyrin repeat of ASPP2 between residues 931–961 [13]. Its binding site overlaps both the binding sites to Bcl-2 and NFκB (Figure 1C and D) [31], as was shown before in peptide array screening [13]. This is possible since the proline rich domain is long and intrinsically disordered [13], and the ASPP2 Ank-SH3 binding sites to Bcl-2 and NFκB are spatially close [31].

### NFκB peptides and p53CD bind overlapping sites in ASPP2 Ank-SH3

Based on our results we suggest that the p53CD binding sites in ASPP2 Ank-SH3 are different from the sites that bind NFκB and Bcl-2. p53CD loop3 (residues 241–249) binds the ASPP2 SH3 domain in the n-src loop and the RT loop (Figure 1A) [14]. The Bcl-2 BH4 peptide (residues 7–24) binds the RT loop of ASPP2 in different residues than the p53 loop3 [14], [22]. In fluorescence anisotropy competition assay the peptides derived from Bcl-2 and NFκB did not displace p53CD from its complex with Fl-ASPP2 n-src peptide. However, p53CD displaced Fl-NFκB 303–332 from its complex with ASPP2 Ank-SH3. These results suggest that NFκB 303–332 binds the ASPP2 SH3 domain mostly through the RT loop (Figure 1B). NFκB 303–332 did not bind the n-src loop peptide in fluorescence anisotropy experiments (data not shown). NFκB 303–332 also competed with the ASPP2 Pro 722–737 peptide that binds ASPP2 via the n-src loop. It is possible that the NFκB 303–332 peptide has an allosteric effect on the binding of the ASPP2 Pro peptide or that its binding to the n-src loop is induced in a cooperative way only after its binding to the RT loop. NFκB 303–332 is also predicted to be intrinsically disordered by different methods [33]–[35], and only NFκB 303–313 is predicted to bind the first ankyrin repeat of ASPP2 [21]. According to the peptide array studies, residues 314–350 in NFκB participate in ASPP2 Ank-SH3 binding [13]. It is thus possible that NFκB 314–332 peptide binds the SH3 domain of ASPP2. Indeed, the NFκB 303–332 peptide (Table 1) contains a proline rich sequence in its C-terminus that could be a ligand of the ASPP2 SH3 domain [36], [37].

ASPP2 and Bbp are both encoded by the TP53BP2 gene but ASPP2 contains 123 additional residues in its N-terminus [8], [11]. NFκB inhibits the pro-apoptotic function of Bbp and prevents cell death by the mitochondrial death pathway. Binding p53 may stabilize Bbp and increase its protein level [38]. It is possible that the pro-apoptotic function of Bbp/ASPP2 (both containing the Ank-SH3 domains) is reduced when NFκB competes with p53 on the interaction with the Ank-SH3 domains. By binding p53, ASPP2 mediates the transactivation action of p53 on pro apoptotic genes [8]. Inhibition of this interaction would thus inhibit the apoptotic function of p53.

**Figure 8:**
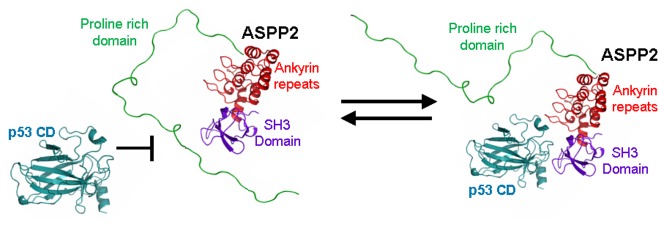
The proposed model [13]: The disordered ASPP2 Pro regulates the intermolecular interactions of ASPP2. The disordered ASPP2 Pro binds to the ASPP2 Ank-SH3 domains and inhibits their protein-protein interactions. Inhibition of the domain-domain interaction makes the Ank-SH3 available to binds its partner proteins such as p53CD.

Our experimental results presented here further support our previous model for the role of the domain-domain interaction in regulating the protein-protein interactions of ASPP2 (Figure 8) [13], [39]. We show that p53CD and the proline rich domain of ASPP2 compete for the same binding sites in ASPP2 Ank-SH3. This sheds light on how ASPP2 regulates p53-mediated apoptosis at the molecular level.
